# Succinate ester derivative of δ-tocopherol enhances the protective effects against ^60^Co γ-ray-induced hematopoietic injury through granulocyte colony-stimulating factor induction in mice

**DOI:** 10.1038/srep40380

**Published:** 2017-02-01

**Authors:** Zhong-Tang Li, Li-Mei Wang, Li-Rong Yi, Chao Jia, Fan Bai, Ren-Jun Peng, Zu-Yin Yu, Guo-Lin Xiong, Shuang Xing, Ya-Jun Shan, Ri-Fang Yang, Jun-Xing Dong, Yu-Wen Cong

**Affiliations:** 1 Department of Pathophysiology, Beijing Institute of Radiation Medicine, Beijing, China; 2 Department of Medicinal Chemistry, Beijing Institute of Pharmacology and Toxicology, Beijing, China; 3 Department of Pharmaceutical Sciences, Beijing Key Laboratory for Radiobiology (BKLRB), Beijing Institute of Radiation Medicine, Beijing, China

## Abstract

α-tocopherol succinate (α-TOS), γ-tocotrienol (GT3) and δ-tocotrienol (DT3) have drawn large attention due to their efficacy as radioprotective agents. α-TOS has been shown to act superior to α-tocopherol (α-TOH) in mice by reducing lethality following total body irradiation (TBI). Because α-TOS has been shown to act superior to α-tocopherol (α-TOH) in mice by reducing lethality following total body irradiation (TBI), we hypothesized succinate may be contribute to the radioprotection of α-TOS. To study the contributions of succinate and to identify stronger radioprotective agents, we synthesized α-, γ- and δ-TOS. Then, we evaluated their radioprotective effects and researched further mechanism of δ-TOS on hematological recovery post-irradiation. Our results demonstrated that the chemical group of succinate enhanced the effects of α-, γ- and δ-TOS upon radioprotection and granulocyte colony-stimulating factor (G-CSF) induction, and found δ-TOS a higher radioprotective efficacy at a lower dosage. We further found that treatment with δ-TOS ameliorated radiation-induced pancytopenia, augmenting cellular recovery in bone marrow and the colony forming ability of bone marrow cells in sublethal irradiated mice, thus promoting hematopoietic stem and progenitor cell recovery following irradiation exposure. δ-TOS appears to be an attractive radiation countermeasure without known toxicity, but further exploratory efficacy studies are still required.

The risk of exposure to ionizing radiation is believed to be increasing due to potential nuclear accidents and terrorist activities. Acute radiation syndrome (ARS) is a collection of health effects presenting within 24 hours of exposure to high doses of ionizing radiation, which is classically divided into three main presentations: hematopoietic, gastrointestinal and neurological/vascular^1^. Radiation countermeasures are defined as agents that prevent, mitigate or treat radiation injury. Several classes of radiation countermeasures have been investigated based on their ability to: (a) reduce free radicals, (b) inhibit apoptosis, and (c) stimulate the hematopoietic system^2^. However, no drug has been approved by Food and Drug Administration (FDA) for use in the treatment against ARS^3^, and as such, natural products with human health benefits have become attractive targets for research. There is significant interest in developing tocols as radioprotectors due to their potent antioxidant properties, and the absence of known side effects^4^,^5^. The term “vitamin E”, a family of eight tocols composed of four isomers, α, β, γ, and δ^6^. Of the tocols, α-tocopherol (α-TOH) has previously been demonstrated to elicit a protective effects against ^60^Co γ-ray-induced hematopoietic injury, with dose reduction factor (DRFs) of 1.06 (1 h before) and 1.11 (15 min after)^7^. Recent studies have shown that α-tocopherol succinate (α-TOS), the hem-isuccinate ester of α-TOH, is far more effective in reducing acute radiation toxicity and preventing radiation-induced lethality^4^,^5^. α-TOS protects mice against radiation-induced hematopoietic syndromes, with a DRF of 1.28 for 30-day survival^8^. Notably, the protective effects of α-TOS against hematopoietic syndromes can be abrogated through the use of a neutralizing antibody targeting granulocyte colony-stimulating factor (G-CSF)^9^,^10^. α-TOS has been demonstrated greater potential than α-TOH in inducing high levels of circulating G-CSF in irradiated and unirradiated animals^11^.

Because α-TOS are superior to α-TOH in reducing lethality following total body irradiation (TBI) and increasing circulating G-CSF in mice, we hypothesized the chemical group succinate may enhance the radioprotective potential of tocopherols. In nature, tocopherols occur in α, β, γ and δ forms, determined by the number and position of methyl groups on the chromanol ring^6^. In the present study, we synthesized α-, γ- and δ-TOS and compared their radioprotective efficacy by determining 30-day efficacy at lethal radiation doses and circulating G-CSF levels in mice. Our results demonstrate that the chemical group succinate significantly affected the radioprotective effects of α-, γ- and δ-TOS and promoted G-CSF stimulation. δ-TOS was a stronger radioprotector among the succinate ester derivatives of tocopherols. δ-TOS treatment ameliorates radiation-induced pancytopenia, augments cellular recovery in bone marrow, improves the colony forming ability of bone marrow cells in sub-lethal irradiated mice, and promotes hematopoietic stem and progenitor cell recovery following irradiation.

## Results

### Synthesis of tocopherol succinate ester derivatives and MTD in mice

**Figure 1:**
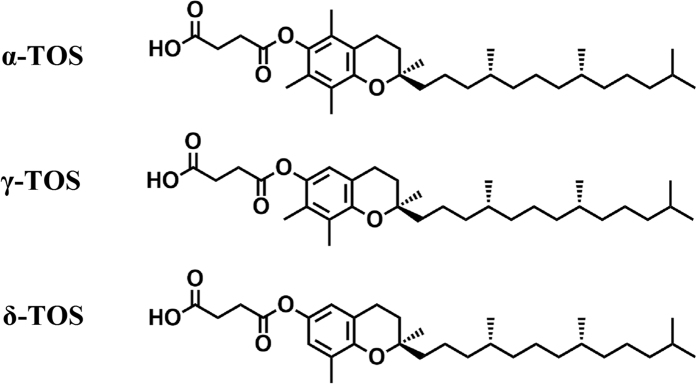
Structure of tocopherol succinates.

α-, γ- and δ-TOS were synthesized by chemical processes, as showed in Figs S1 and S5. The structures are shown in Fig. 1, which were identified by HPLC-MS and H^1^-NMR. The acute toxicity of these compounds was studied for 14 days following post-subcutaneous administration. Single doses up to 1500 mg/kg injected subcutaneously (s.c.) were well tolerated by the mice, and no deaths were noted.

### Tocopherol derivatives protecte mice from lethal doses of gamma radiation

In our previous experiment, α-TOS, a known radioprotector, provided maximum protection against radiation when administered s.c. at dose of 400 mg/kg 24 h prior to and 1 h post-radiation exposure^5^. According to the two-dose schedule of α-TOS, we compared the radioprotective properties of α-, γ- and δ-TOS in irradiated mice.

**Figure 2:**
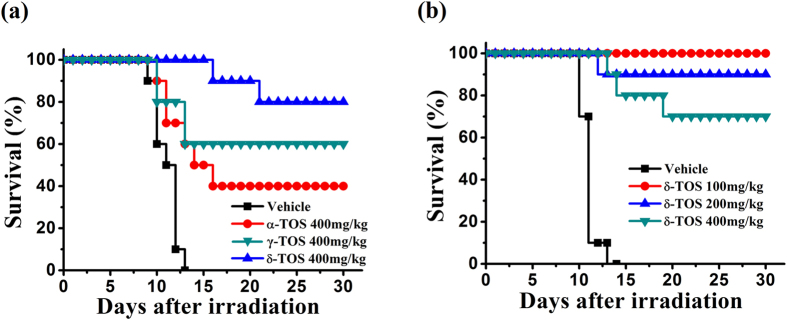
Tocopherol derivatives protected mice from lethal doses of gamma radiation. (**a**) Kaplan-Meier survival rate curve of 4 groups (n = 10) administrated with PEG400 (vehicle), α-TS (400 mg/kg), γ-TOS (400 mg/kg), δ-TOS (400 mg/kg) 24 h before and 1 h after the 9.5 Gy TBI. (**b**) Kaplan-Meier survival rate curve of 4 groups (n = 10) administrated with PEG400 (vehicle), δ-TOS (100 mg/kg), δ-TOS (200 mg/kg), δ-TOS (400 mg/kg) 24 h before and 1 h after the 9.5 Gy TBI. P value was calculated by log-rank test.

In preliminary experiments, mice were irradiated with a dose of 9.0 Gy TBI for 30 days to assess survival. This dose of radiation has been shown to induce severe bone marrow myelosuppression and is associated with a high incidence of mortality. As showed in Fig. S2, 10% of the mice receiving PEG-400 vehicle alone survived 18 days post-irradiation. Treatment with α-, γ- and δ-TOS resulted in significant (P < 0.01) radioprotection of wild-type mice compared to vehicle-treated controls, with a 60–90% survival benefit over vehicle-treated controls. To better define the radioprotective efficacy for each tocopherols and perform a comparative analysis to the effects mediated by α-TOS, we increase TBI to 9.5 Gy. At this dose, all vehicle mice died 10 days post-irradiation. We found that treatment with α-, γ- and δ- TOS conferred 40%, 60% and 80% survival against the 9.5 Gy dose of TBI (Fig. 2a), whereas δ-TOS treatment elicited a 80% survival advantage in mice. This finding significantly different when compared to the 40% survival advantage found in the group that received α-TOS treatment. These results demonstrate that δ-TOS confer greater protection against radiation when delivered to mice before and after TBI.

To determine the optimal radioprotection dose, mice were irradiated with a dose of 9.5 Gy TBI and different doses of δ-TOS were administered s.c. 24 h prior to, and 1 h following radiation exposure. As shown in Fig. 2b, all mice in the vehicle arm of this study that received 9.5 Gy by 13 days post-irradiation. However, 100 mg/kg of δ-TOS resulted in 100% 30-day survival, while increasing the dose to 200 mg/kg and 400 mg/kg showed 80% and 70% 30-day survival. Based on these results, 100 mg/kg was the optimal radioprotective dose of δ-TOS and selected for use in subsequent experiments.

### δ-TOS strongly accelerates hematological recovery post-irradiation

Hematopoietic cells are the highly sensitive to ionizing radiation and, as such, irradiated mice often die from hematopoietic failure. Radiation-induced bone marrow injury results in loss of hematopoietic cells and consequent leukopenia and thrombocytopenia^12^. To understand how δ-TOS protects mice against lethal irradiation, we monitored the recovery of peripheral blood cells following sub-lethal irradiation with 6.5 Gy, a dose of radiation sufficient to allow full recovery from radiation-induced depletion of peripheral blood in 30 days.

**Figure 3:**
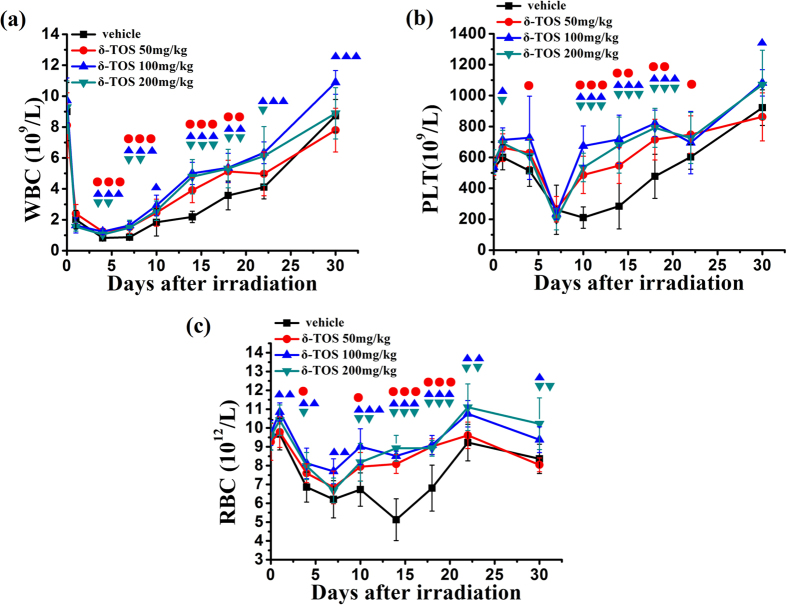
δ-TOS accelerates hematological recovery post-irradiation. (**a**) The white blood cells count, (**b**) the platelets count, (**c**) the red blood cells count in 30 days after the 6.5 Gy TBI of 4 groups administrated with PEG400 (vehicle), δ-TOS (50 mg/kg), δ-TOS (100 mg/kg), δ-TOS (200 mg/kg) 24 h before and 1 h after the 6.5 Gy TBI. Data represent the mean ± S.E.M. (n = 8). ●, ▲, ▼ P < 0.05; ●●, ▲▲, ▼▼ P < 0.01; ●●●, ▲▲▲, ▼▼▼ P < 0.001 vs. control (vehicle) group.

As showed in Fig. 3a and c, a severe radiation-induced depletion of blood cell counts was observed in mice shortly after 6.5 Gy TBI. Administration of δ-TOS at 50 mg/kg, 100 mg/kg and 200 mg/kg 24 h prior to and 1 h following radiation exposure corresponded to a more robust recovery of white blood cells (WBC), red blood cells (RBC) and platelets over a 30 day time period, demonstrating an enhanced hematopoietic recovery in irradiated mice. Similar to the result observed during the 30-day survival period, 100 mg/kg was the optimal radioprotective dose of δ-TOS to elicit hematological recovery post-irradiation. Doubling the dose to 200 mg/kg, did not further increase the efficacy of treatment, while halving the dose to 50 mg/kg markedly decreased the efficacy as compared to a dose of 100 mg/kg (P < 0.01).

In an effort to characterize whether δ-TOS would elicit a protective effect against radiation *in vivo*, much like α-TOS. The effects of δ-TOS, administered at dose of 100 mg/kg 24 h prior to or 1 h following TBI at a single dose or combination of both on radiation-induced cytopenia were observed. As showed in Supplemental Fig. S3, a single dose of δ-TOS administered at 24 h before or 1 h after radiation exposure resulted in a significant recovery of RBC and platelets over a 30 day time period. This effect was more pronounced when administered 24 h before radiation, indicating δ-TOS is an effective radiation protector when delivered to mice before or after TBI. Moreover, combination of the two treatments resulted in the most robust WBC, RBC and platelet recovery, which exhibited superiority from that of mice treated with a single dose of δ-TOS 1 h after TBI at days 14.

### δ-TOS augmented cellular recovery in bone marrow and enhanced the colony forming ability of bone marrow cells in sublethal irradiated mice

**Figure 4:**
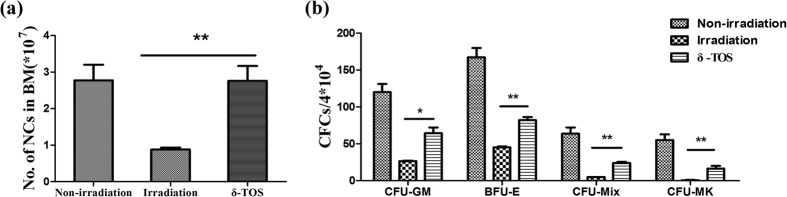
Effects of δ-TOS on nucleated cells of marrow and colony-forming assay. Non-irradiated group was administrated vehicle without irradiation. Irradiated group and δ-TOS group were respectively administrated with vehicle and δ-TOS (100 mg/kg) 24 h before & 1 h after 6.5 Gy TBI. (**a**) Bone marrow nuclear cells (BMNCs) count of each group on the 10th day after irradiation. (**b**) Colony-forming unit-granulocyte and monocyte (CFU-GM) count, burst-forming unit-erythroid (BFU-E) count, burst forming unit-megakaryocyte (BFU-MK) count and mixed colony-forming unit (CFU-Mix) count on the 10th day after irradiation. Data represent the mean ± S.E.M. (n = 8); *P < 0.05; **P < 0.01 vs. irradiated group.

To understand how δ-TOS facilitates hematological recovery post lethal irradiation, we studied the hematopoietic recovery in bone marrow. 100 mg/kg of δ-TOS was administered to mice 24 h before and 1 h after 6.5 Gy TBI. Bone marrow cells were collected from femora on day 10 following irradiation and total live bone marrow myeloid cells for pooled samples from each mouse were measured by trypan blue staining. All irradiated groups exhibited fewer bone marrow mononuclear cells than unirradiated, age-matched normal controls. However, δ-TOS treated mice had significantly more bone marrow mononuclear cells than any other irradiated cohort at days 10 (Fig. 4a). To further understand the effect of δ-TOS on hematopoiesis, we also measured hematopoietic progenitor cells using a CFU assay. As shown in Fig. 4b, total clonogenic activity in the marrow compartment (CFU-GM, BFU-E, CFU-GEMM and CFU-Mix) was markedly reduced 10 days after TBI, and δ-TOS significantly increased the frequency of colony forming cells (CFC) in all lineages.

**Figure 5:**
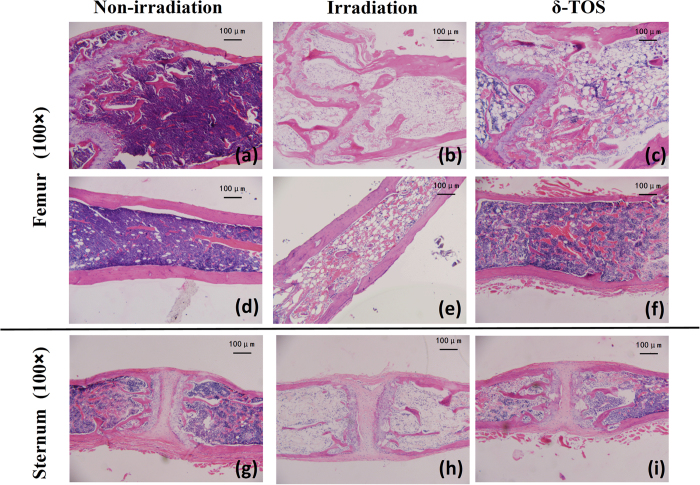
Effects of δ-TOS on bone marrow recovery after sublethal irradiation. Non-irradiated group was administrated vehicle without irradiation. Irradiated group and δ-TOS group were respectively administrated with vehicle and δ-TOS (100 mg/kg) 24 h before & 1 h after 6.5 Gy TBI. Hematoxylin and eosin staining of the BM cavities of femurs and sternums: (**a** and **d**) Femurs from non-irradiated group; (**b** and **e**) Femurs from irradiated group; (**c** and **f**) Femurs from δ-TOS group; (**g**) Sternums from non-irradiated group; (**h**) Sternums from irradiated group; (**i**) Sternums from δ-TOS group.

To further analyze the mechanism by which δ-TOS contributes to hematological recovery post-irradiation, histological analysis of bone marrow of 6.5 Gy irradiated mice were performed on Day 7, which represented the day of estimated maximal bone marrow suppression based on the timing of nadir of blood cell counts. As shown in Fig. 5, an examination of longitudinal femoral and sternebrae bone marrow sections from vehicle mice demonstrated marked hypocellularity, cytolysis, fatty tissue and cell debris increases, and sinusoid blood congestion, all of which are indicative of severe radiation-induced marrow depletion. In contrast, the femora and sternebrae from δ-TOS-treated animals demonstrated mostly mineralized trabeculae and a few regenerating pockets/foci of hematopoietic cells, indicating a significant hematopoietic recovery in bone marrow.

### δ-TOS promotes hematopoietic stem and progenitor cell recovery after irradiation exposure

**Figure 6:**
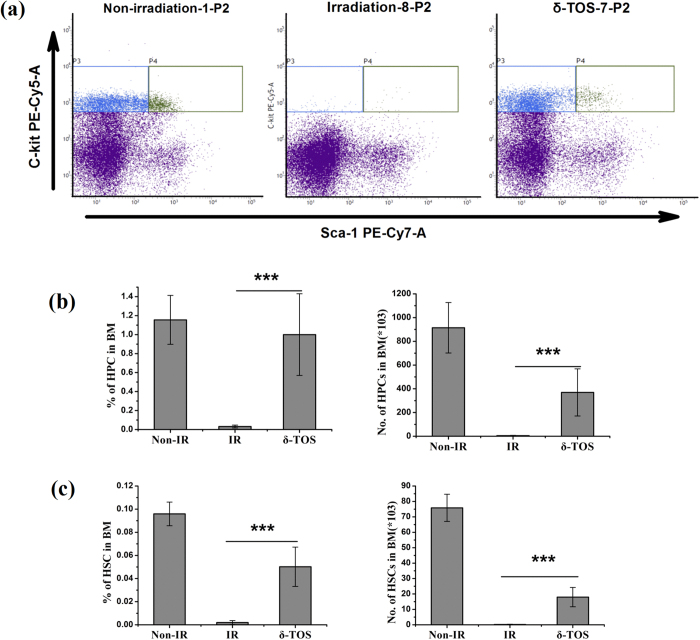
δ-TOS promotes hematopoietic stem and progenitor cell recovery after irradiation exposure. Non-irradiated group (Non-IR) was administrated vehicle without irradiation. Irradiated group (IR) and δ-TOS group were respectively administrated with vehicle and δ-TOS (100 mg/kg) 24 h before & 1 h after 6.5 Gy TBI. (**a**) Representative flow cytometry of hematopoietic progenitor cells (HPCs, c-Kit+Lin−Sca1−) and hematopoietic stem cells (HSCs, c-Kit+Lin−Sca1+) in the BM of each group. Shown in P2, sca1 and c-Kit were gated on Lin− cells. (**b**) Quantifications and frequencies of HPCs in whole bone marrow cells on days 10 after irradiation. (**c**) Quantifications and frequencies of HSCs in whole bone marrow cells on days 10 after irradiation. Data represent the mean ± S.E.M. (n = 8); ***P < 0.001 vs. irradiated group.

It is well established that hematopoietic stem and progenitor cells (HSPCs) play predominant roles in radioprotection^13^. We assessed HSPCs repopulation in bone marrow of 6.5 Gy irradiated mice on day 10 after irradiation. The frequencies of hematopoietic stem (LSK, *Lin*^−^ *Sca-1*^+^ *c-Kit*^+^) and progenitor (LK, *Lin*^−^ *Sca-1*^−^ *c-Kit*^+^) were determined by flow cytometric assays (Fig. S4). As showed in Fig. 6a–c, the percentage and total number of hematopoietic stem (LSK) and progenitor (LK) cells decreased markedly by day 10 following TBI. However, δ-TOS treated mice showed a higher percentage of LSK and LK cells among the total BM cells, which resulted in a 75-fold and 80-fold increase in the absolute number of LSK and LK cells compared with that in vehicle mice.

**Figure 7:**
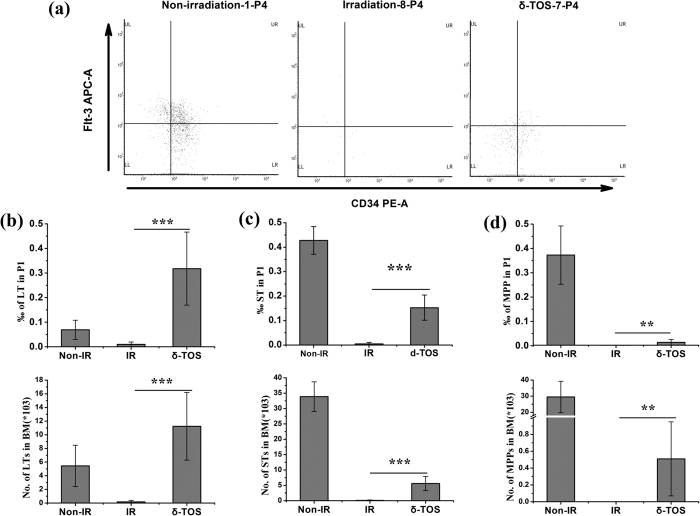
δ-TOS promotes long term and short term hematopoietic stem cell recovery after irradiation exposure. Non-irradiated group (Non-IR) was administrated vehicle without irradiation. Irradiated group (IR) and δ-TOS group were respectively administrated with vehicle and δ-TOS (100 mg/kg) 24 h before & 1 h after 6.5 Gy TBI. (**a**) Representative flow cytometry of LT-HSCs cells, ST-HSCs cells and MPP-HSCs in the BM of each group on days 10 after irradiation. Shown in P4, flt-3 and CD34 were gated on LSK cells. (**b**) Quantifications and frequencies of LT-HSCs cells in whole bone marrow cells on days 10 after irradiation. (**c**) Quantifications and frequencies of ST-HSCs cells in whole bone marrow cells on days 10 after irradiation. (**d**) Quantifications and frequencies of MPP-HSCs cells in whole bone marrow cells on days 10 after irradiation. Data represent the mean ± S.E.M. (n = 8); **P < 0.01; ***P < 0.001 vs. irradiated group.

To determine the effects of δ-TOS on HSCs, we further analyzed the frequencies of long-term hematopoietic stem cells (LT-HSC, CD34^**−**^LSK cells), short term hematopoietic stem cells (ST-HSC, CD34^+^LSK cells) and multi-potent progenitors (MPP, Flt3^+^CD34^+^ LSK cells) in BM 10 days after 6.5 Gy TBI. As showed in Fig. 7a–d, the percentage and the total number of ST-HSC, ST-HSC and MPP decreased markedly by day 10 after TBI. Notably, δ-TOS treatment promoted the recovery of these cells in BM after TBI in comparison with vehicle treatment. More importantly, the percentage as well as the absolute number of ST-HSC in δ-TOS treated irradiated mice were similar or some higher than the normal level. These results demonstrate that δ-TOS significantly improves the recovery of hematopoietic stem and progenitor cells following radiation injury.

### Induction of G-CSF by tocopherol succinate ester derivatives in mice

**Figure 8:**
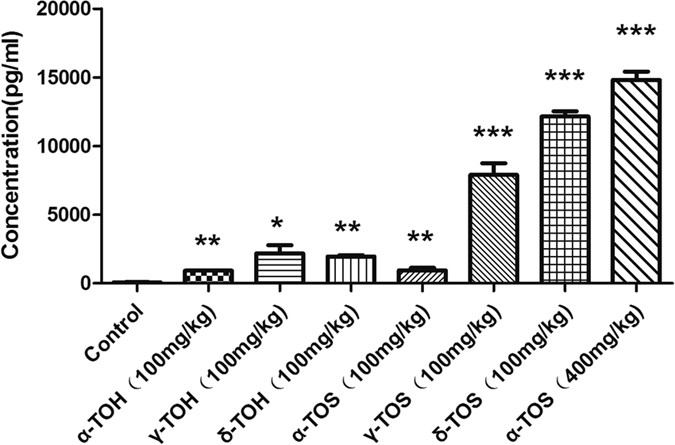
Effect of of tocopherol and its derivatives on G-CSF production. Quantification of the G-CSF in serum of the mice with respective treatment of PEG400 (vehicle), α-TOH, γ-TOH, δ-TOH, α-TOS, γ-TOS and δ-TOS. Data represent the mean ± S.E.M. (n = 5); *P < 0.05; **P < 0.01; ***P < 0.001 vs. control (vehicle) group.

The ongoing studies with different radiation countermeasures such as 5-AED, genistein, toll-like receptor agonists as well as γ-tocotrienol (GT3), δ-tocotrienol (DT3) and α-TOS support the hypothesis that the protection of mice from radiation-induced hematopoietic death is mediated by G-CSF induction^14^,^15^,^16^,^17^,^18^,^19^. We therefore compared the levels of G-CSF following the administration of α-, γ- and δ -TOH as well as their succinate ester derivatives. 24 h after administration of drug or vehicle, serum samples were collected and analyzed using mouse G-CSF ELISA kit. As showed in Fig. 8, a 100-mg/kg dose of α-, γ- and δ-TOH as well as α-TOS resulted in a 15 to 35-fold increase in G-CSF induction compared to vehicle. In contrast, the succinate ester derivatives of γ- and δ-TOH administrated at the dose of 100-mg/kg resulted in a 130 to 200-fold increase in G-CSF induction compared to vehicle, coinciding with their survival efficacy against ionizing radiation. As reported in previous studies, α-TOS at the optimal dose of 400-mg/kg resulted in a 245-fold increase in G-CSF induction compared to vehicle. These results demonstrate that succinate ester derivatives of tocopherol especially δ-TOS strongly enhanced G-CSF induction in mice.

## Discussion

In recent years, natural compounds or nutraceuticals with human health benefits have generated considerable interest as a potential radiation countermeasure^2^,^3^. Both tocopherols and tocotrienols are collectively known as tocols, each have four analogs (*α, β, γ*, and *δ*). Among analogs, α-TOS, GT3 and DT3 have drawn the most attention as they provide better radioprotective efficacy^4^,^5^,^20^,^21^. Because α-TOS is superior to α-tocopherol in reducing lethality in mice and increasing circulating G-CSF following TBI, we hypothesized succinate may enhance the radioprotective potential of tocopherols. In the present study, α-, γ- and δ- TOS were synthesized and their radioprotective efficacy and G-CSF induction in mice were compared. The results of our study validate our hypothesis, confirming that δ-TOS is the strongest radioprotector among the succinate ester derivatives of tocopherols.

The radioprotective properties of tocols have been demonstrated during the past decade. α-TOH (100 IU/kg) administered subcutaneously either 1 h before or 15 min after γ-irradiation significantly increased the 30-day survival of mice, with DRFs of 1.06 and 1.11^7^. Higher subcutaneous doses of α-TOH (400 IU/kg) enhanced the survival of irradiated mice when given 24 h before *γ*-irradiation^22^. α-TOS, the hemi-succinate ester derivative of *α*-TOH, is more effective than *α*-TOH in reducing lethality after TBI. In fact, a single dose of α-TOS (400 mg/Kg), given 24 hours before ^60^Co *γ*-radiation exposure can protect mice from acute doses of *γ*-radiation with a DRF of 1.28^8^. *α*-tocopherol-monoglucoside (*α*TMG) and *γ*-tocopherol-N,N-dimethylglycine ester (GTDMG) are water-soluble derivatives of tocopherol. It was previously reported that intraperitoneal injection of *α*TMG (600 mg/Kg) or GTDMG (100 mg/Kg) immediately after irradiation was radioprotective, with a DRF of 1.09 and 1.25^23^,^24^. GT3 and DT3 have gained prominence in recent years as they provide better radioprotective efficacy. When administered 24 h prior to ^60^Co radiation, single doses of GT3 (200 mg/Kg) or DT3 (300 mg/Kg) protected mice from death following lethal levels of radiation, with a DRF of 1.29 and 1.27^25^,^26^. Herein, we compared the radioprotective potential of the succinate ester derivatives of tocopherol, and found δ-TOS is a strongest radioprotector among the succinate ester derivatives of tocopherols, which conferred 80% survival against the 9.5 Gy dose of TBI, whereas γ-TOS and α-TOS treatment elicited a 60% and 40% survival advantage, respectively. We determined the optimal radioprotection dose was 100 mg/kg of δ-TOS, which resulted in a 100% 30-day survival against the 9.5 Gy dose of TBI, while increasing the dose to 200 mg/kg and 400 mg/kg showed 80% and 70% 30-day survival. With these factors in mind, δ-TOS appears to be an attractive radiation countermeasure candidate with higher radioprotective efficacy at a lower dose of administration.

α-TOS, GT3 and DT3 have been reported to induce high levels of G-CSF. Using a neutralizing antibody against G-CSF, studies have shown that levels of G-CSF induced by all three agents are directly related to the degree of protection against TBI^9^,^10^,^18^,^19^. α-TOS has been shown to induce high levels of G-CSF, which peaks at 24 h following administration, thus corresponding to the optimal radioprotection when α-TOS is given 24 h before TBI^9^,^10^. Therefore, G-CSF may represent a biomarker of the radioprotective effects of these promising radiation countermeasures. Confirming previously published studies^11^, we found α-, γ- and δ-TOH as well as α-TOS administered at a dose of 100-mg/kg induced lower levels of G-CSF. In contrast, the succinate ester derivatives of γ- and δ-TOH administrated at the dose of 100-mg/kg resulted in higher level of G-CSF, which is also higher than that observed following administration of α-TOS at the optimal dose of 400-mg/kg. These results demonstrated that succinate ester derivatives of tocopherols especially δ-TOS and γ-TOS induce a high level of GCSF to confer stronger protection.

Exposure to radiation has been demonstrated to lead to lymphopenia, neutropenia, thrombocytopenia and anemia due to the damage of the hematopoietic system. Exposed subjects, if left unattended, succumb to hemorrhage and infection^12^,^13^. δ-TOS administered to mice 24 h before and/or 1 h after 6.5 Gy TBI has been found to ameliorated leucopenia, thrombocytopenia as well as anemia, indicating that it has very specific effects on the trilineage compartments in peripheral blood. There was also an associated increase hematopoietic stem (LSK) and progenitor (LK), as well as long-term hematopoietic stem cells (LT-HSC), short term hematopoietic stem cells (ST-HSC) and multi-potent progenitors (MPP) in BM 10 days after 6.5 Gy TBI. Also noted were higher cellularity in the bone marrow and an increased number of colony-forming units of bone marrow cells. It has been reported that radiation exposure increases the circulating level of G-CSF and that administration of a neutralizing antibody to G-CSF exacerbates the deleterious effects of ionizing radiation and abrogates the radioprotective efficacy of some potential radiation countermeasures such as α-TOS, GT3 and DT3^9^,^10^,^18^,^19^,^27^. Consistent with the notion that hematopoietic cell preservation and recovery could be explained by the higher serum G-CSF levels after δ-TOS treatment.

The exact mechanism by which tocols and their derivatives induce radioprotection or G-CSF induction remains poorly understood. The most commonly described mechanism of action of these agents results from their function as antioxidants^4^,^5^. In contrast, the radioprotective efficacy of a tocol is not determined solely by its antioxidant activity. For example, *α*-TOH has been shown to have better antioxidant activity than GT3, DT3, or α-TOS. However, the radioprotective efficacy of these three tocols does not follow the same trend^4^,^5^,^6^,^28^. α-TOS, GT3 and DT3 have been shown to demonstrate a strong apoptogenic activity against many cancer cell lines^6^. Naturally occurring derivatives of tocopherol, including α-, γ- and δ-TOH, were largely non-apoptogenic. Succinylation induces pro-apoptotic activity, although the activity of γ- and δ-TOS was lower than that of α-TOS^29^. As indicated by the present study, there is some correlation between the radioprotective efficacy or G-CSF induction of tocopherol succinate ester derivatives and their apoptogenic activity against many cancer cells. This is the case of α-tocotrienol, which, unlike GT3 and DT3, is non-apoptogenic in a variety of malignant cells, and also not report to have radioprotective efficacy. Ionizing radiation, employed in radiotherapy for almost 40% of cancer patients, results not only in the destruction of cancer cells, but also damages the normal cells^30^. As such radioprotection of normal cells and the antitumor activity of some tocols and their derivatives support their specific use in cancer patients receiving radiotherapy^4^,^5^,^6^.

Overall, our findings indicate that succinylation of α-, γ- and δ-TOH enhances the radioprotective efficacy and G-CSF induction of the agents in mice and found δ-TOS is a strongest radioprotector among the succinate ester derivatives of tocopherol. δ-TOS has the potential to serve as both radioprotectors and radiomitigators based on its ability to induce G-CSF, to protect mice from radiation-induced lethality, as well as to accelerate hematopoietic recovery in irradiated mice. As compared to previous studies with α-TOS, δ-TOS seems to be a more attractive radiation countermeasure candidate without known toxicity. Recently, GT3 has demonstrated radioprotective efficacy in the nonhuman primate (NHP) model. At 5.8 and 6.5 Gy total-body irradiation, GT3 treatment completely prevented thrombocytopenia^31^. Further research is needed to elucidate the molecular mechanisms by which δ-TOS induces its radioprotective effect, and evaluate efficacy in non-human primates to strengthen the pre-clinical data and establish parameters for a phase I clinical trial.

## Materials and Methods

### Agents, Antibodies and Equipments

Tocopherols were purchased from Sigma (St. Louis. MO). Succinic anhydride, hexane and PEG400 were purchased from Sinopharm Chemical Reagent Co.,Ltd. RayBio^®^ Mouse G-CSF ELISA kit was purchased from RayBiotech Inc. PE-Cy5 conjugated Anti-mouse CD117 (C-kit) antibody (anti-c-kit-PE-Cy5) and PE-Cy7 conjugated Anti-mouse Ly-6A/E (Sca-1) antibody (anti-Sca-1-PE-Cy7) were obtained from eBioscience Inc. FITC conjugated anti-mouse Lineage (CD3/Gr-1/CD11b/CD45R(B220)) antibody (anti-Lin-FITC), PE conjugated anti-mouse CD34 antibody (anti-CD34-PE), APC conjugated anti-mouse CD135 (Flt-3) (anti-Flt-3-APC) antibody and anti-mouse CD16/32 antibody (anti-CD16/32) were obtained from Biolegend Inc. MethoCult GF 3434 StemSpan media was from StemCell Technologies (Vancouver, BC, Canada). Celltac E Auto-Hematology-Analyzer (MEK-7222K) was purchased from Nihon Kohden Corporation (Tokyo, Japan) with relative reagents supplied by Nihon Kohden Inc. (Shanghai, China). A BD FACSVerse^TM^ Flow cytometer for cell analysis and sorting was purchased from Becton Dcikinson Inc.

### Synthesis of Tocopherol Succinates

According to the method reported by Smith *et al*.^29^, α, γ- and δ-tocopherol succinate were synthesized. The general procedure for this preparation is shown in Fig. S1. In brief, a solution of tocopherol (5 mmol) in hexane was added to succinic anhydride (7.5 mmol) and triethylamine (0.5 mmol), and the mixture was refluxed for 10 h. After tocopherol was totally reacted, the mixture was cooled to room temperature and concentrated under vacuo. The residue was purified by flash chromatography using hexane/ethyl acetate (6:1) to give the desired products as white solids. The structure shown in Fig. 1 was demonstrated by HPLC-MS and H^1^-NMR (Fig. S5).

### Mice

SPF C57BL/6 J male mice between 8~10 weeks of age and weighting 20~24 g were purchased from Beijing HFK Bioscience Co. Ltd. The animals were kept in Laboratory Animal Center, the Academy of Military Medical Sciences for one week prior to the initiation of this study. The mice had free access to pellet food and water and were housed at a temperature of 22 ± 2 °C and relative humidity of 50~60% on a 12-h light/dark cycle. Animal welfare and experimental procedures were carried out in accordance to National Institutes of Health guidelines for the care and use of laboratory animals. This study was approved by Beijing Experimental Animal Ethics Committee (2006) No. 5118 set by the Beijing People’s Government. Animals in this study were sacrificed by cervical dislocation. All efforts were made to minimize discomfort, distress, pain and injury.

### Radiation Treatment

Irradiation studies were performed using a ^60^Co irradiator Sciences at a dose rate of 51.3 ± 0.2 cGy/min at the Beijing Institute of Radiation Medicine. Mice were set into an organic glass box for the total-body irradiation (TBI) and then kept in the barrier system of Laboratory Animal Center at the Academy of Military Medical Sciences. The dose of irradiation in the survival rate evaluation was 9.0 Gy and 9.5 Gy, the dose in the other experiments was 6.5 Gy.

### Survival Rate Evaluation of the Lethal Doses γ-Ray Irradiated Mice

Briefly, 40 mice were randomly divided into four equal groups and were subcutaneously administrated either PEG400 (vehicle), α-TOS (400 mg/kg), γ-TOS (400 mg/kg), or δ-TOS (400 mg/kg) 24 h before and 1 h after irradiation. Following exposure to 9.0 Gy TBI, the mice were kept for 30 days to evaluate overall survival.

To better study the radioprotective efficacy of different tocopherol succinates, the same procedure described above was repeated with 40 mice exposed to 9.5 Gy TBI. For an optimal administration-dose study, another 40 mice were equally divided into four groups administrated with PEG400 (vehicle), δ-TOS (100 mg/kg), δ-TOS (200 mg/kg), δ-TOS (400 mg/kg) 24 h before and 1 h after the 9.5 Gy TBI.

### Analyzing the Peripheral Blood of the sublethal γ-Ray Irradiated Mice

To study the effect of δ-TOS on peripheral blood cells of irradiated mice, 32 mice were equally divided into four groups at random, and subcutaneously administrated either PEG400 (vehicle), δ-TOS (50 mg/kg), δ-TOS (100 mg/kg), or δ-TOS (200 mg/kg) at both 24 h before and 1 h after the exposure to 6.5 Gy TBI. On the 1^st^, 4^th^, 7^th^, 10^th^, 14^th^, 18^th^, 22^th^ and 30^th^ days following irradiation, 20 μl blood was collected via the caudal vein and added with 2 ml blood cell diluents. Every sample was mixed uniformly prior to analysis using a Celltac E Auto-Hematology-Analyzer. Curves of white blood cells (WBC), red blood cells (RBC) and platelets count were depicted.

For an optimal administration-time study, another four groups with 8 mice each were exposed to 6.5 Gy TBI. Three groups were administrated with δ-TOS at a single dose of 100 mg/kg 24 h before, 1 h after and 24 h before & 1 h after irradiation and the control group was administrated with PEG400 (vehicle) 24 h before & 1 h after irradiation. The samples were collected and analyzed as described above.

### Bone Marrow Nuclear Cells (BMNCs) Count and Colony Forming Unit (CFU) Assay

To study the number of bone marrow cells following irradiation, 12 mice were randomly divided into three groups (n = 4). Two groups were respectively administrated with PEG400 (vehicle) and δ-TOS (100 mg/kg) 24 h before & 1 h after 6.5 Gy TBI and the other group was administrated with PEG400 (vehicle) at the same time interval, but without irradiation. On the 10^th^ day following irradiation, all mice were sacrificed by cervical dislocation and immersed in alcohol (75%) for 5 min. Femora on both sides were excised and cleaned of adhering muscle tissue. To obtain a single-cell suspension of bone marrow, the bone marrow was flushed out with 1 ml RPMI-1640 medium. 50 μl was for the BMNCs count and the left was resuspended at 5 × 10^4^ cells/ml in MethoCult GF 3434 StemSpan media for the colony forming unit assay. 12-well plates with 1 ml dilution per well were kept for 7 days in a cell culture box of 5% CO_2_ at 37 °C. Colony-forming unit-granulocyte and monocyte (CFU-GM), burst-forming unit-erythroid (BFU-E), burst forming unit-megakaryocyte (BFU-MK) and mixed colony-forming unit (CFU-Mix) were counted and statistically analyzed.

### Bone Marrow Staining

Mice were randomly separated into three groups (n = 3 per group), of which two were administrated with PEG400 (vehicle) and δ-TOS (100 mg/kg) 24 h before & 1 h after 6.5 Gy TBI and the other one was administrated with PEG400 (vehicle) at the same time interval but without irradiation. On the 7^th^ day post-irradiation, mice were sacrificed via cervical dislocation, and femora and sterna were excised and cleaned of adhering tissues. Following fixation in formaldehyde solution (10%), decalcification in formic acid (5%), dehydration in alcohol, paraffin embedding and section, Hematoxylin and eosin (H&E) staining assay was performed as the standard procedure.

### Flow Cytometric Analysis

As described above, BMMCs were obtained by flushing the femur, and the cells were resuspended in 100 μl PBS buffer. 1 ml Red Blood Cell Lysis Buffer was added and allowed to incubate for 10 min at room temperature. Cells were passed through 45 μM cell strainer and washed twice with PBS prior to staining with an CD16/32 antibody and with anti-c-kit-PE-Cy5, anti-Sca-1-PE-Cy7, anti-Lin-FITC, anti-CD34-PE and anti-Flt-3-APC. Stained cells were washed twice with PBS and re-suspended in RPMI-1640 medium. Analysis and sorting were performed on a BD FACSVerseTM Flow cytometer.

### ELISA

ELISA assay to measure the G-CSF level in blood was performed using a RayBio^®^ Mouse G-CSF ELISA kit, in accordance with manufacturer instructions. Eight groups of mice (n = 5) were respectively administrated PEG400 (vehicle), α-TOH (100 mg/kg), γ-TOH (100 mg/kg), δ-TOH (100 mg/kg), α-TOS (100 mg/kg), α-TOS (400 mg/kg), γ-TOS (100 mg/kg), δ-TOS (100 mg/kg). Twenty-four hours following administration of the agents, the mice were anesthetized by intraperitoneal administration of sodium pentobarbital and blood samples were collected from heart. Serum for the measurement was obtained after blood clotting and centrifugation.

### Statistics

All data represent mean ± SEM and the Graph Pad Prism 5.02 was used for statistical analyzing. Results comparison analyzing was including two-tailed Student’s t-test for two groups and one-way Analysis of Variance (ANOVA) for multiple comparisons to control group. Survival was analyzed by Kaplan-Meier analysis with the long-rank test. Statistical significance was defined as p < 0.05.

## Additional Information

**How to cite this article**: Li, Z.-T. *et al*. Succinate ester derivative of δ-tocopherol enhance the protective effects against ^60^Co γ-ray-induced hematopoietic injury through granulocyte colony-stimulating factor in mice. *Sci. Rep.* **7**, 40380; doi: 10.1038/srep40380 (2017).

**Publisher's note:** Springer Nature remains neutral with regard to jurisdictional claims in published maps and institutional affiliations.

## Supplementary Material

Supplemental Material
