# Supplementary material for: Succinate ester derivative of δ-tocopherol enhances the protective effects against 60Co γ-ray-induced hematopoietic injury through granulocyte colony-stimulating factor induction in mice
Source: Sci Rep. 2017 Feb 1;7:40380. doi: 10.1038/srep40380 (PMC5286428; doi:10.1038/srep40380)
Supplement: Supplemental Material [file srep40380-s1.pdf]

SUPPLEMENTAL MATERIAL

Succinate ester derivative of δ-tocopherol enhances the protective effects against ^{60}Co γ-ray-induced hematopoietic injury through granulocyte colony-stimulating factor induction in mice

Zhong-Tang Li [a#], Li-Mei Wang [a#], Li-Rong Yi [#], Chao Jia [a], Fan Bai [a], Ren-Jun Peng [a], Zu-Yin Yu [a], Guo-Lin Xiong [a], Shuang Xing [a], Ya-Jun Shan [a], Ri-Fang Yang [c], Jun-Xing Dong [b*], Yu-Wen Cong [a*]

[a] Department of Pathophysiology, [b] Department of Pharmaceutical Sciences, Beijing Key Laboratory for Radiobiology (BKLRB), Beijing Institute of Radiation Medicine, Beijing, China

[c] Department of Medicinal Chemistry, Beijing Institute of Pharmacology and Toxicology, Beijing, China

# These authors contributed equally to the study.

*Corresponding author: Yu-Wen Cong, Department of Pathophysiology, Beijing Institute of Radiation Medicine, No. 27 Taiping Road, Beijing 100850, China, and Jun-Xing Dong, Department of Medicinal Chemistry, Beijing Institute of Radiation Medicine, No. 27 Taiping Road, Beijing 100850, China

Tel.: +86 10 68210077 Ext. 931223; fax +86 10 68214653; e-mail: congyw@nic.bmi.ac.cn

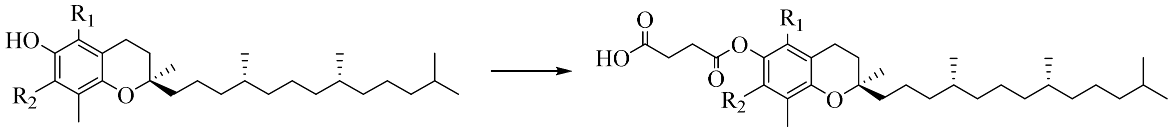

α -TOS: R_1=methyl; R_2=methyl
γ -TOS: R_1=H; R_2=methyl
δ -TOS: R_1=H; R_2=H

Reagents and conditions: Succine anhydride, hexane, TEA, refluxing, 10h, 85-94%.

Supplemental Figure 1. syntheses of tocopherol succinates

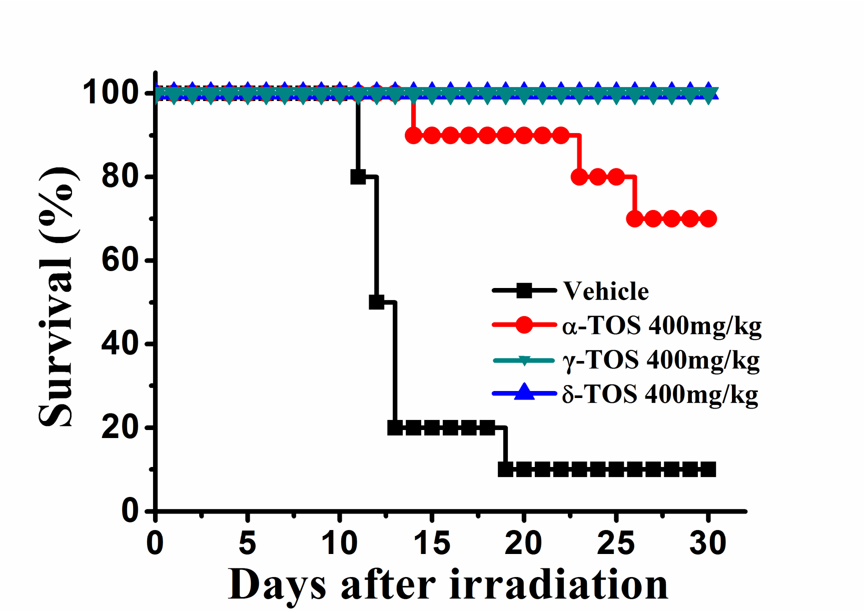

Supplemental Figure 2. Tocopherol derivatives protected mice from the 9.0 Gy TBI.

Kaplan-Meier survival rate curve of 4 groups (n=10) respectively administrated with PEG400 (vehicle), α-TS (400 mg/kg), γ-TOS (400 mg/kg) and δ-TOS (400 mg/kg) 24 h before and 1 h after the 9.0 Gy TBI. *P* value was calculated by log-rank test.

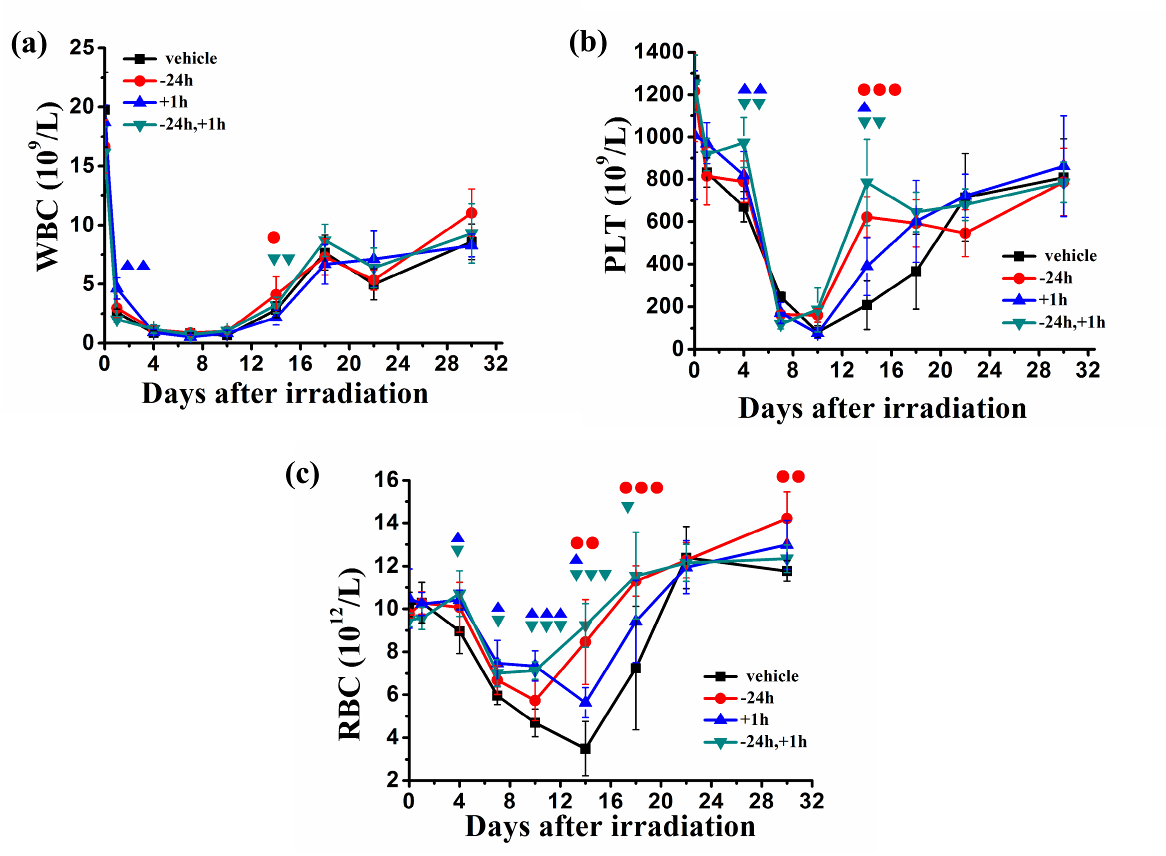

Supplemental Figure 3. Radioprotection and mitigation of δ-TOS against radiation induced hematopoietic injury. (**a**) The white blood cells count, (**b**) the platelets count, (**c**) the red blood cells count in 30 days after the 6.5 Gy TBI of 3 groups (n=8) respectively administrated with δ-TOS (100 mg/kg) 24 h before, 1 h after, and 24 h before ＆ 1 h after the irradiation. The control group was administrated with PEG400 (vehicle) 24 h before ＆ 1 h after the irradiation. Data represent the mean±S.E.M. (n=8); ●P, ▲P, ▼P<0.05; ●●P , ▲▲P,▼▼P＜0.01; ●●●P , ▲▲▲P, ▼▼▼P< 0.001 *vs.* control (vehicle) group.

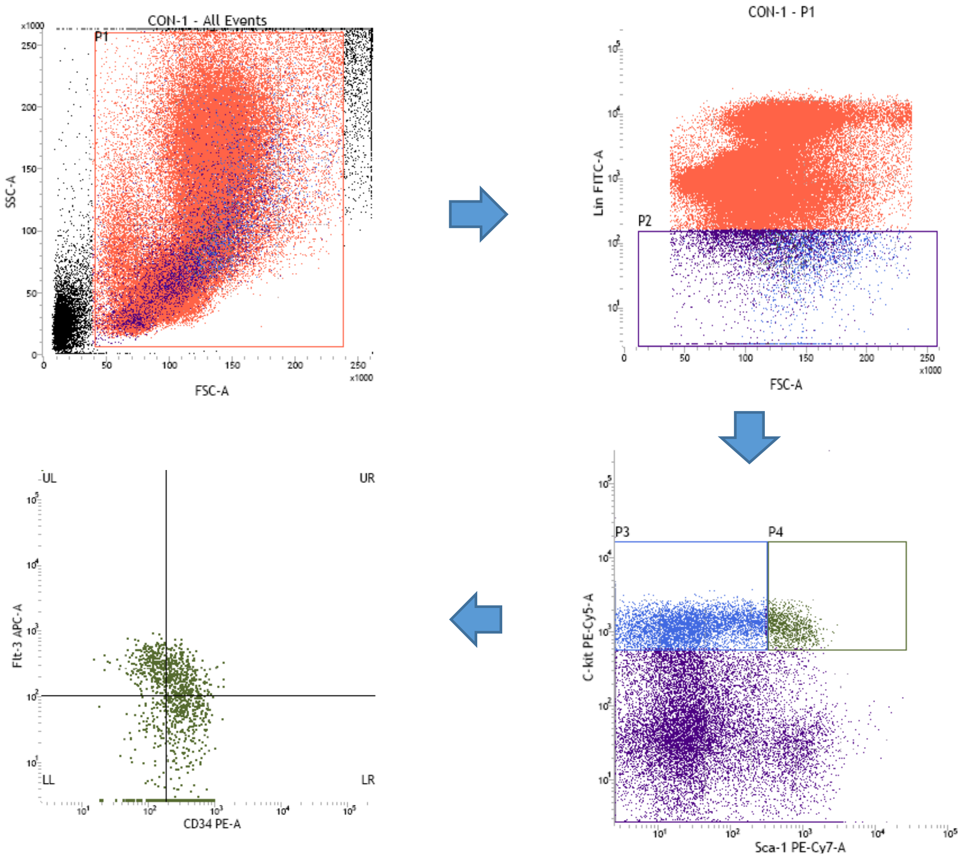

Supplemental Figure 4. Flow cytometric assays of hematopoietic stem and progenitor cell of mouse bone marrow cells

Supplemental Figure 5.

α-TOS

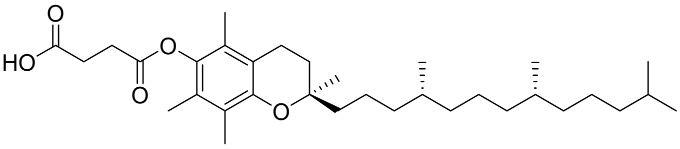

Chemical Formula: $C_{33}H_{54}O_5$
Molecular Weight: 530.79

H^1-NMR

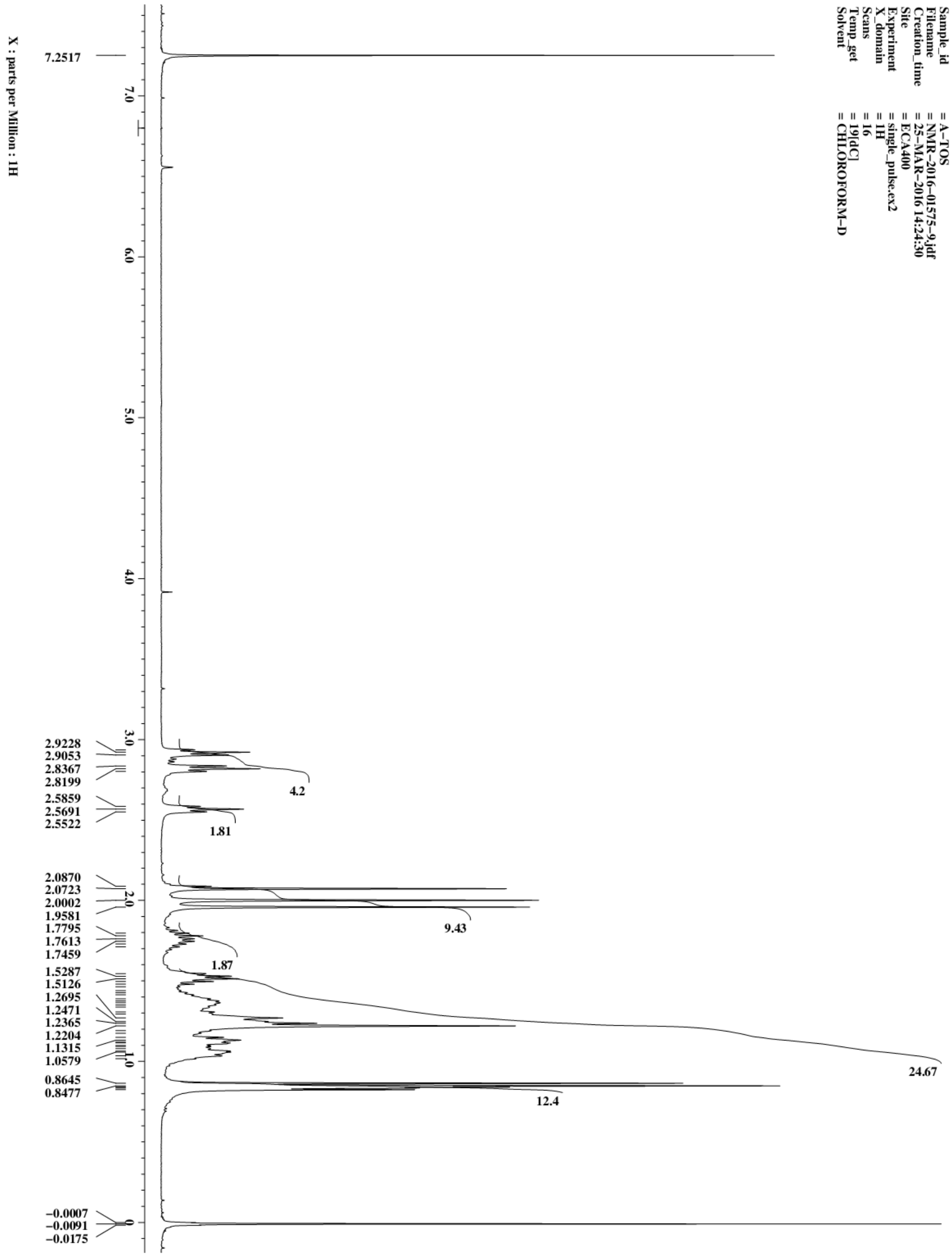

HPLC-MS

Qualitative Analysis Report

**Data Filename**	0907.d	**Sample Name**	A-TOS
**Instrument Name**	TOF G6230A	**Acquired Time**	2016-03-25
**Acq Method**	YCL.M	**Acquired SW**	6200 series TOF/6500 series
**IRM Calibration Status**	**Success**		

User Chormatograms

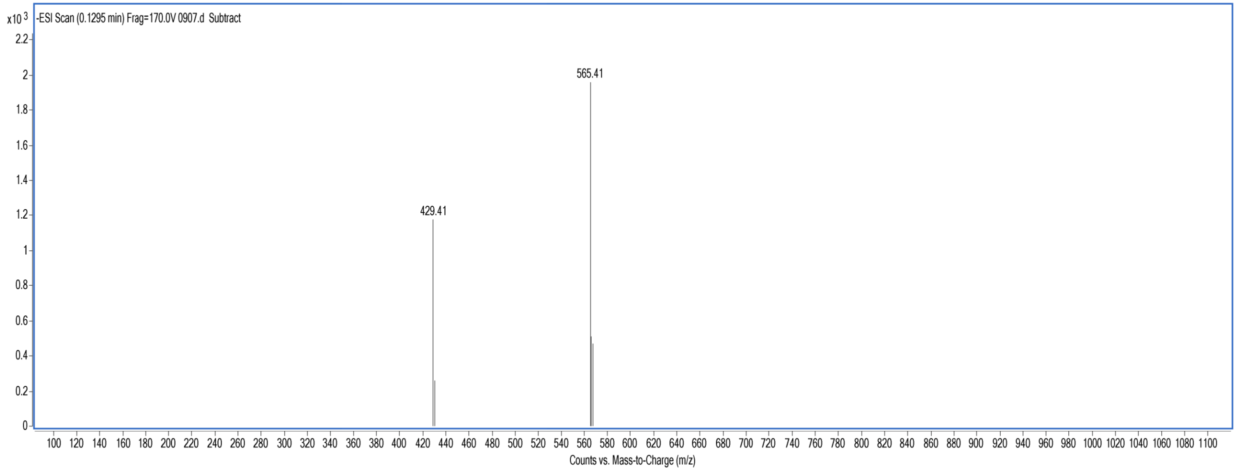

γ-TOS

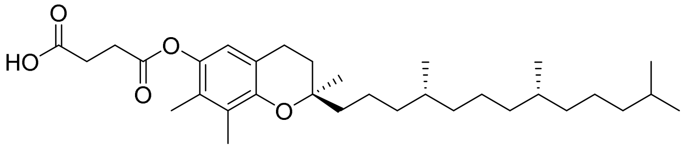

Chemical Formula: $C_{32}H_{52}O_5$
Molecular Weight: 516.76

H[1]-NMR

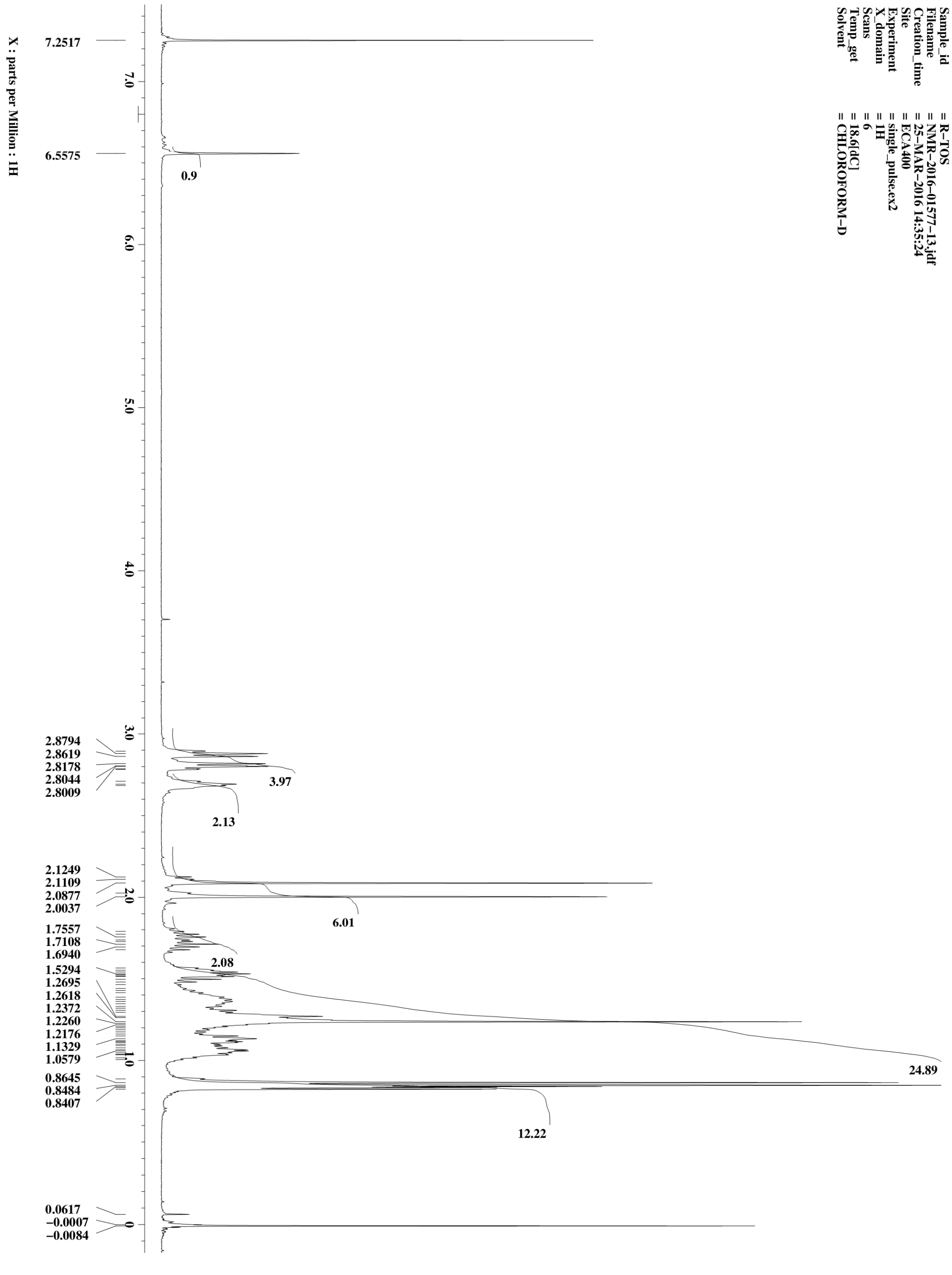

HPLC-MS

Qualitative Analysis Report

**Data Filename**	0908.d	**Sample Name**	R-TOS
**Instrument Name**	TOF G6230A	**Acquired Time**	2016-03-25
**Acq Method**	YCL.M	**Acquired SW**	6200 series TOF/6500 series
**IRM Calibration Status**	**Success**		

User Chormatograms

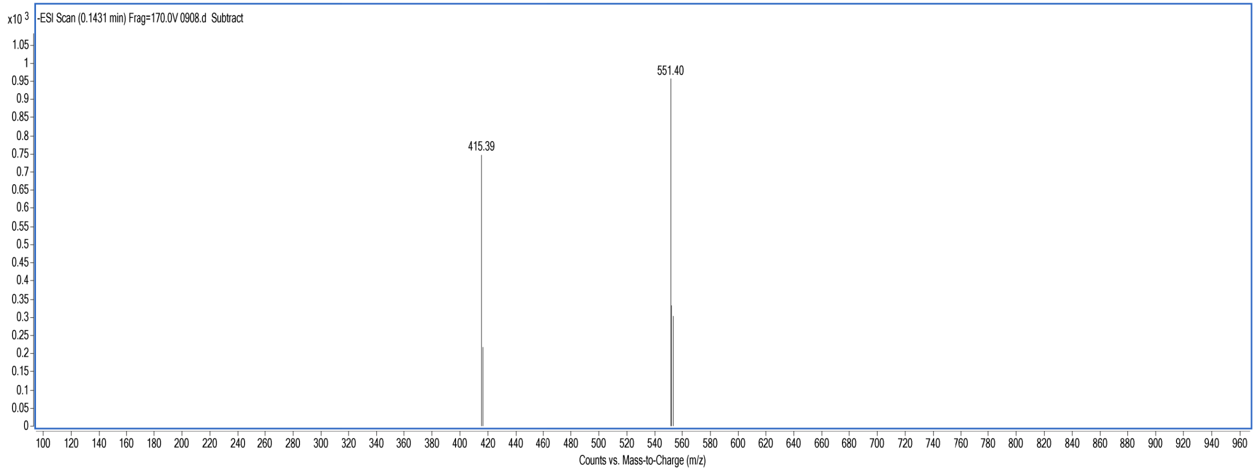

δ-TOS

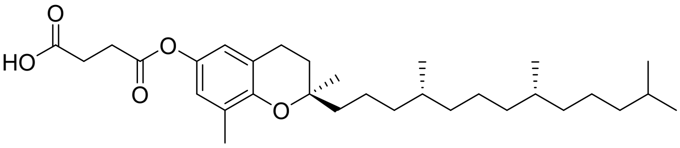

Chemical Formula: $C_{31}H_{50}O_5$
Molecular Weight: 502.74

H^1-NMR

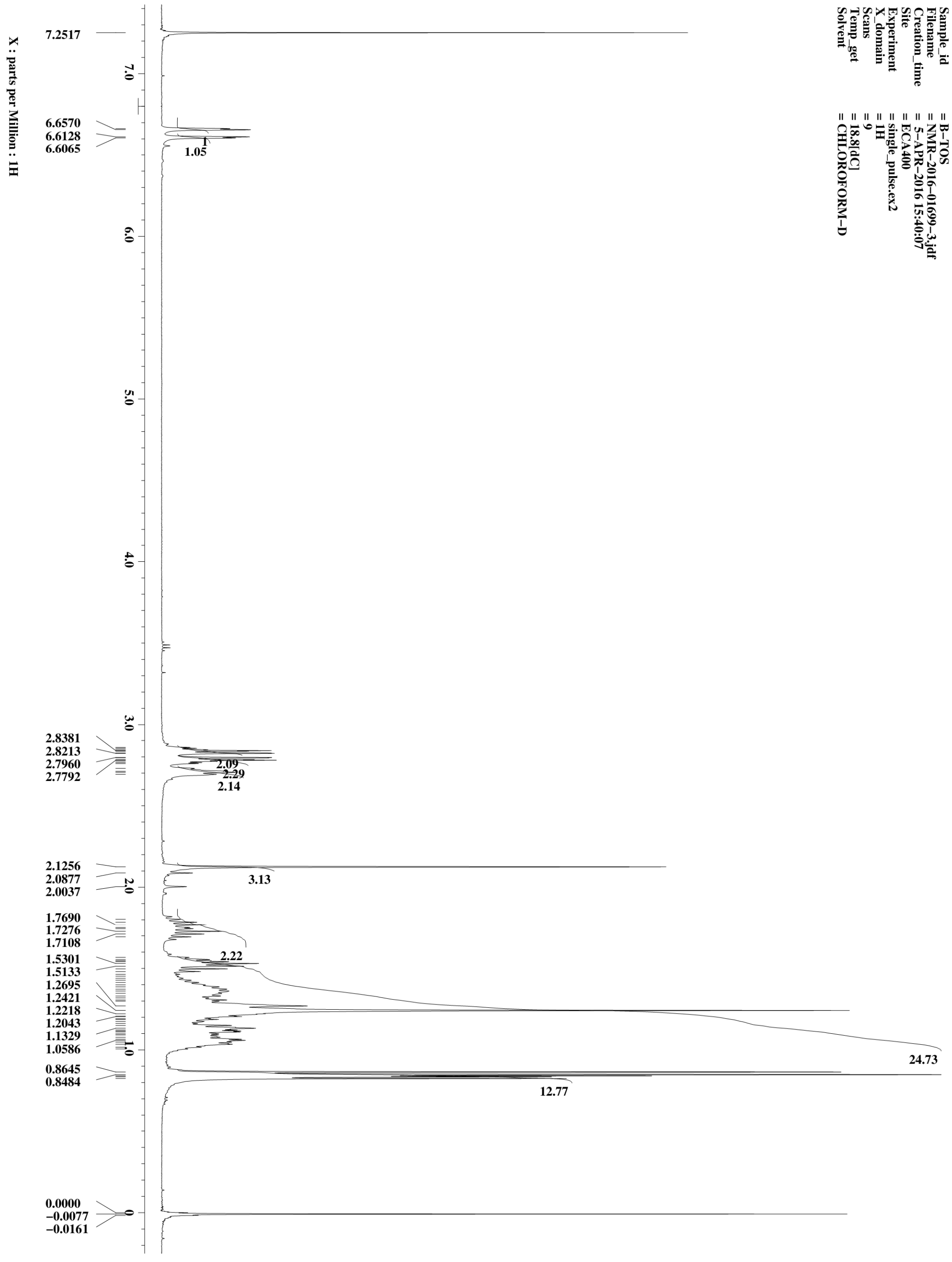

δ-TOS HPLC-MS

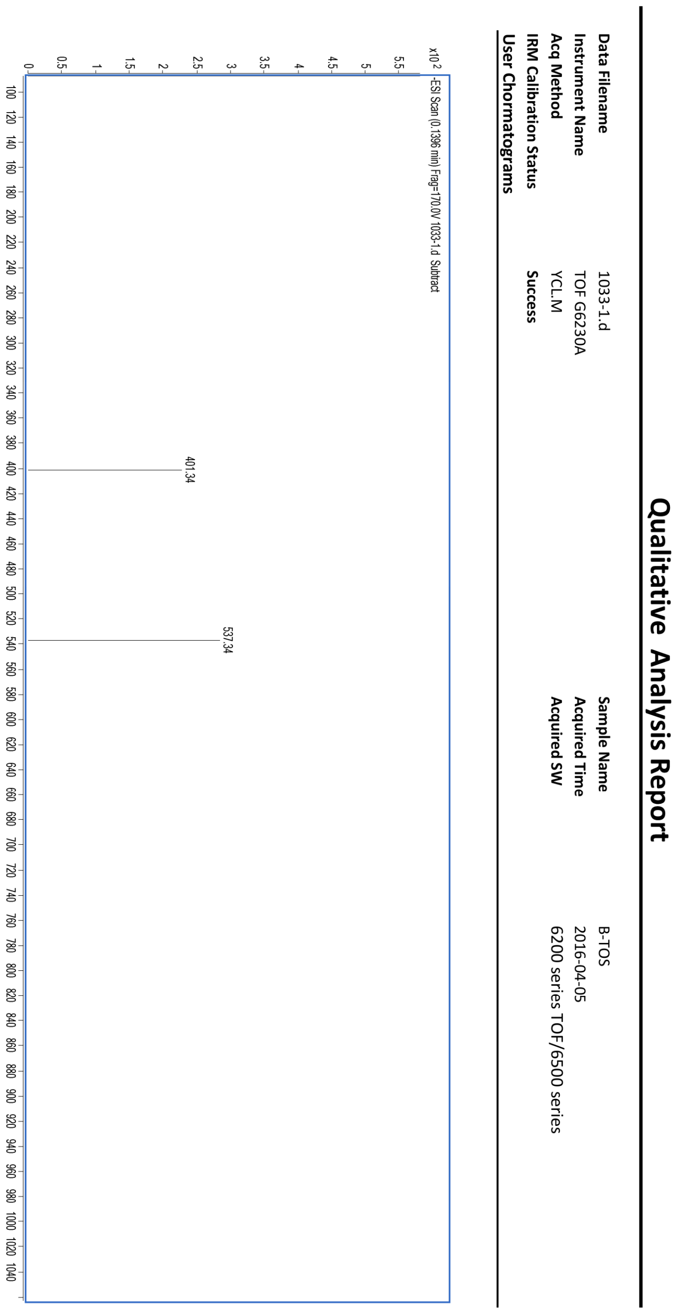

Supplemental Figure 5. HPLC-MS and δ-TOS H^1-NMR
